# Clinical Characteristics of COVID-19 Patients With Gastrointestinal Symptoms: An Analysis of Seven Patients in China

**DOI:** 10.3389/fmed.2020.00308

**Published:** 2020-06-09

**Authors:** Jin-Wei Ai, Hao Zi, Yong Wang, Qiao Huang, Na Wang, Lu-Yao Li, Bin Pei, Jianguang Ji, Xian-Tao Zeng

**Affiliations:** ^1^Evidence-Based Medicine Center and The Third Ward of Orthopedic, Xiangyang No. 1 People's Hospital, Hubei University of Medicine, Xiangyang, China; ^2^Center for Evidence-Based and Translational Medicine, Zhongnan Hospital of Wuhan University, Wuhan, China; ^3^Department of Radiology, Xiangyang No. 1 People's Hospital, Hubei University of Medicine, Xiangyang, China; ^4^Institute of Evidence-Based Medicine and Knowledge Translation, Henan University, Kaifeng, China; ^5^School of Nursing and Health Sciences, Henan University, Kaifeng, China; ^6^Center for Primary Health Care Research, Lund University/Region Skåne, Malmö, Sweden

**Keywords:** SARS-CoV-2, COVID-19, 2019-nCoV, digestive tract, diarrhea, gastrointestinal symptom

## Abstract

**Objectives:** Patients with novel coronavirus disease 2019 (COVID-19) can present with gastrointestinal symptoms as their initial symptoms or as the main manifestations during disease progression, but the clinical characteristics of these patients are still unknown.

**Methods:** We identified COVID-19 patients who admitted to Xiangyang No. 1 People's Hospital and presented with gastrointestinal symptoms as their initial or main symptoms. Their medical records were reviewed by two independent clinical scientists. The epidemiological and clinical characteristics as well as the clinical outcomes were analyzed.

**Results:** Among 142 confirmed COVID-19 cases, 7 (4.9%) of them presented with gastrointestinal symptoms. Three patients had gastrointestinal symptoms as the initial symptoms and chief complaints, and 4 patients as the main symptoms during disease progression. Six patients had symptoms of diarrhea (3–16 days), 7 with anorexia (7–22 days), 6 with upper abdominal discomfort (1–7 days), and 4 with nausea (1–7 days), 1 with heartburn lasting 2 days, and 2 with vomiting symptoms (1 day). The chest CT scan showed typical COVID-19 imaging features, and associated with the progression of the disease. During treatment, 2 patients died due to organ failure.

**Discussion:** COVID-19 patients with gastrointestinal symptoms are relatively rare and might be misdiagnosed. The clinical features include watery stools, anorexia, and upper abdominal discomfort. These patients may have severe disease and be associated with a poor prognosis. The underlying mechanisms of SARS-CoV-2 related gastrointestinal symptoms need to clarify in future studies.

## Introduction

In December 2019, patients with pneumonia of unknown cause appeared in Wuhan, Hubei, China, and then quickly spread to many provinces and even other countries in a short time (1). Genetic analysis using deep sequencing analysis from patients' respiratory tract specimens showed that the disease was caused by a novel coronavirus named severe acute respiratory syndrome coronavirus 2 (SARS-CoV-2) (2, 3). The World Health Organization named the pneumonia caused by SARS-CoV-2 as a novel coronavirus disease (COVID-19), and announced that COVID-19 was a Public Health Emergency of International Concern (3–5).

People at all ages were susceptible to SARS-CoV-2, especially middle-aged and elderly (6, 7). The main symptoms were fever, cough, shortness of breath, and other respiratory symptoms (8, 9). However, recent data suggest that few COVID-19 cases might present with gastrointestinal symptoms as the initial symptoms (chief complaint), or as the main manifestations during disease progression (1, 10). It should be noted that COVID-19 cases with gastrointestinal symptoms may have a missed or delayed diagnosis, leading to unnecessary transmission of SARS-CoV-2 (11, 12). However, such data are still lacking. It is, therefore, necessary to explore the clinical and epidemiological manifestations of COVID-19 cases with gastrointestinal symptoms to understand the underlying causes, and the disease progression. As of April 9, 2020, a total of 142 patients with COVID-19 were treated at Xiangyang No. 1 People's Hospital, China, of which 7 patients had gastrointestinal symptoms as their main clinical manifestations. This study summarized their epidemiological and clinical manifestations, as well as the associated clinical outcomes.

## Methods

### Study Design and Patients

This was a retrospective study conducted in the Xiangyang No. 1 People's Hospital. The diagnostic criteria of COVID-19 were in accordance with the protocol published by the National Health Commission and the National Administration of Traditional Chinese Medicine, and a real-time reverse-transcriptase–polymerase-chain-reaction (RT-PCR) was used to detect positive nucleic acid of SARS-CoV-2 (1, 9, 13, 14). We defined patients with gastrointestinal symptoms as having diarrhea, nausea, vomiting, heartburn, and upper abdominal discomfort as their initial symptoms or as the main manifestations during the course of the illness. The patient must have the gastrointestinal symptoms for more than 3 days and the complete course of the disease for more than 21 days. Since anorexia was not a specific symptom, patients with only anorexia were not considered in this study. Patients with COVID-19 who had a digestive system disease before admission, and patients who were critically ill at the time of admission were also excluded. This study was reviewed and approved by the Medical Ethical Committee of Xiangyang No. 1 People's Hospital on February 12, 2020 (approval number: 2020GCP012). Written, informed consent was obtained from the individuals for the publication of any potentially identifiable images or data included in this article.

### Data Collection

The baseline data included sex, age, and comorbidities. Epidemiological data included travel history and history of close contact with COVID-19 cases. Clinical data included initial symptoms (chief complaint), main gastrointestinal symptoms and duration, other symptoms, chest X-ray, chest CT scan, laboratory examination, treatment, and outcomes. All data were collected separately and cross-checked by two researchers. Independent review of the chest CT results was done by two senior radiologists, and disagreement was resolved after discussion. After the patients were admitted, three members of the COVID-19 expert team in Xiangyang No. 1 People's Hospital conducted consultations and developed treatment protocols. Patients with obvious dyspnea, dysfunction of other organs, or those requiring life support treatment were transferred to the intensive care unit. Data were collected up to April 9, 2020.

### Statistical Analysis

All data were descriptive statistics and image processing was performed using GraphPad Prism 7.0 software.

## Results

As of April 9, 2020, a total of 542 suspected and confirmed COVID-19 patients were admitted in the Xiangyang No. 1 People's Hospital. There were 142 patients who met the COVID-19 diagnostic criteria. Among the 142 COVID-19 cases, 7 patients were finally included in the study (P1–P7) after excluding patients with only anorexia and those with gastrointestinal symptoms lasting <3 days. Of the 7 patients, 4 were male and 3 were female, and the age ranged from 35 to 75 years. One patient had a history of uterine fibroids (without surgery) and anemia (Table 1). Three patients had a history of travel to Wuhan, 2 patients had a history of close contact with Wuhan residents, and 1 patient's family members were diagnosed with COVID-19. The incubation period for SARS-CoV-2 was 2–9 days.

**Table 1:** Baseline and epidemiological characteristics of COVID-19 patients with gastrointestinal symptoms.

**Patient code**	**Sex**	**Age (years)**	**Comorbidity**	**Exposure history**	**Incubation period (days)**	**Initial symptom/chief complaint**	**Main gastrointestinal symptoms**	**Other symptoms**	**Disease severity**	**Clinical outcome**
P1	Female	54	Urticarial	Contact with Wuhan residents	11 (January 14–25)	Diarrhea and fever for 1 week	Diarrhea, anorexia, nausea, vomiting, upper abdominal discomfort	Fever, cough, headache, shortness of breath, choking sensation in chest, dyspnea	Severe	Recovery
P2	Female	48	Uterine fibroids; anemia	Contact with infected case	8 (January 24–February 1)	Fever for 3 days; diarrhea for 1 day	Diarrhea, anorexia, nausea, vomiting, upper abdominal discomfort	Fever, cough, fatigue, myalgia, shortness of breath, choking sensation in chest	Regular	Recovery
P3	Female	50	None	Recently visited Wuhan	7 (January 22–28)	Anorexia and intermittent fever for 1 week	Anorexia, diarrhea, nausea, upper abdominal discomfort	Fever, fatigue	Regular	Recovery
P4	Male	35	None	Recently visited Wuhan	8 (January 19–27)	Cough and shortness of breath for 3 days	Heartburn, anorexia, upper abdominal discomfort	Fever, cough, shortness of breath	Regular	Recovery
P5	Male	42	None	Recently visited Wuhan	9 (January 22–31)	Fever for 3 days	Diarrhea, anorexia, nausea	Fever, cough, running nose	Regular	Recovery
P6	Male	75	None	Unclear	Unclear	Fever for 3 days	Diarrhea, anorexia, upper abdominal discomfort	fever, cough, sputum production, fatigue, myalgia, dyspnea, shortness of breath, choking sensation in chest	Critical	Critically ill (day 18), death (day 35)
P7	Male	75	None	Contact with Wuhan residents	2 (January 18–20)	Fever for 4 days	Diarrhea, anorexia, upper abdominal discomfort	Fever, cough, sputum production, fatigue, dyspnea, shortness of breath	Critical	Death (day 23)

**Figure 1:**
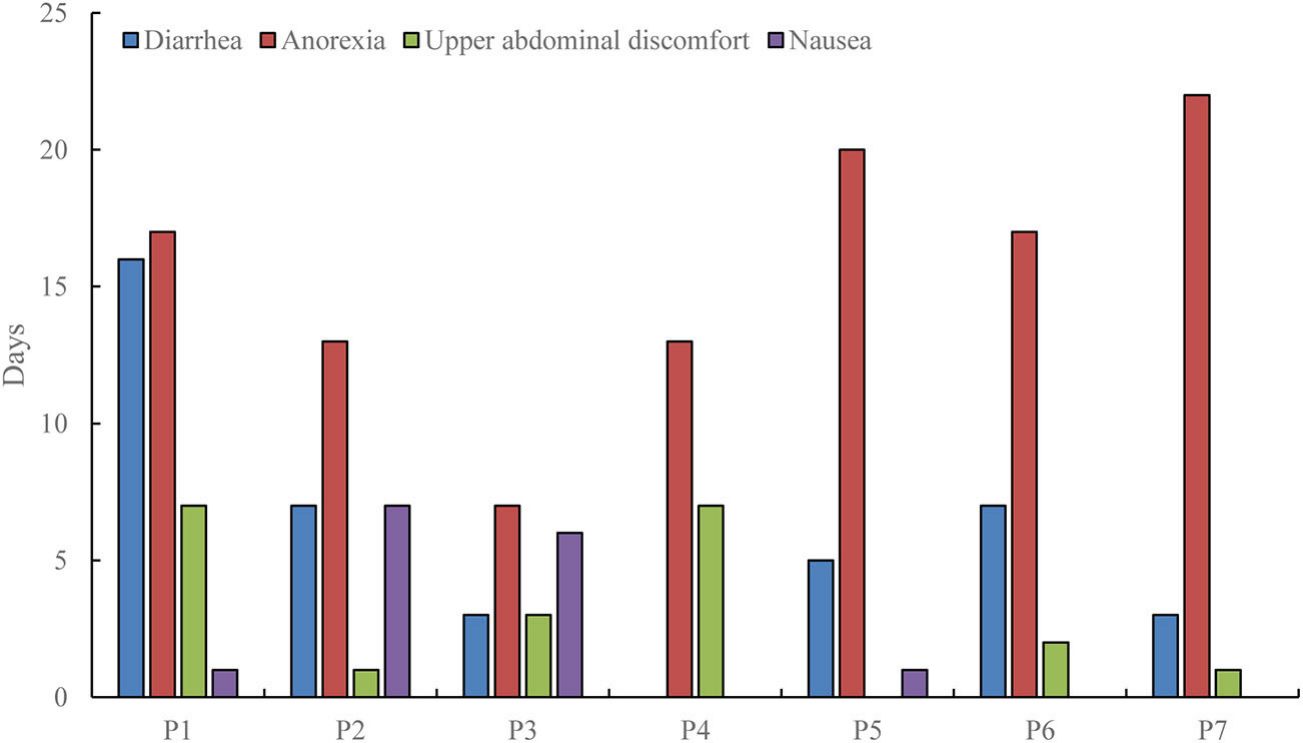
Main gastrointestinal symptoms and duration of COVID-19 patients.

Three patients were hospitalized with gastrointestinal symptoms as the chief complaint, and 4 patients had severe gastrointestinal symptoms during the course of the illness. In addition, 6 patients had symptoms of diarrhea (3–16 days), 7 with anorexia (7–22 days), 6 with upper abdominal discomfort (1–7 days), and 4 with nausea (1–7 days), 1 with heartburn (gastric burning sensation) lasting for 2 days, and 2 with vomiting symptoms (1 day). All patients underwent physical examination and no positive signs of digestive system were found. The main gastrointestinal symptoms and duration were shown in Figure 1, while other symptoms and duration were shown in Table 2.

**Table 2:** Other symptoms and duration of COVID-19 patients.

**Duration of symptoms (days)**	**P1**	**P2**	**P3**	**P4**	**P5**	**P6**	**P7**
Diarrhea	16	7	3	0	5	7	3
Anorexia	17	13	7	13	20	17	22
Upper abdominal discomfort	7	1	3	7	0	2	1
Heartburn	0	0	0	2	0	0	0
Nausea	1	7	6	0	1	0	0
Vomiting	1	1	0	0	0	0	0
Fever	10	8	4	1	5	6	8
Cough	8	6	0	12	13	1	8
Sputum production	8	0	0	0	0	1	0
Running nose	0	0	0	0	3	0	0
Headache	8	0	0	0	0	0	0
Fatigue	1	7	7	0	0	17	22
Myalgia	1	1	0	0	0	3	0
Choking sensation in chest	14	1	0	0	0	2	0
Shortness of breath	19	2	0	15	0	2	1
Dyspnea	11	0	0	0	0	12	18

The chest CT scan showed typical COVID-19 imaging features, including focal nodules, patchy shadows or ground glass opacity. White lung appearance and multiple consolidations of the mediastinum window were shown in severe cases. One patient had emphysema without obvious pleural effusion. The typical progression of chest CTs among patient 1 and 7 was shown in Figure S1, and the remaining chest CTs were shown in Figure S2.

**Figure 2:**
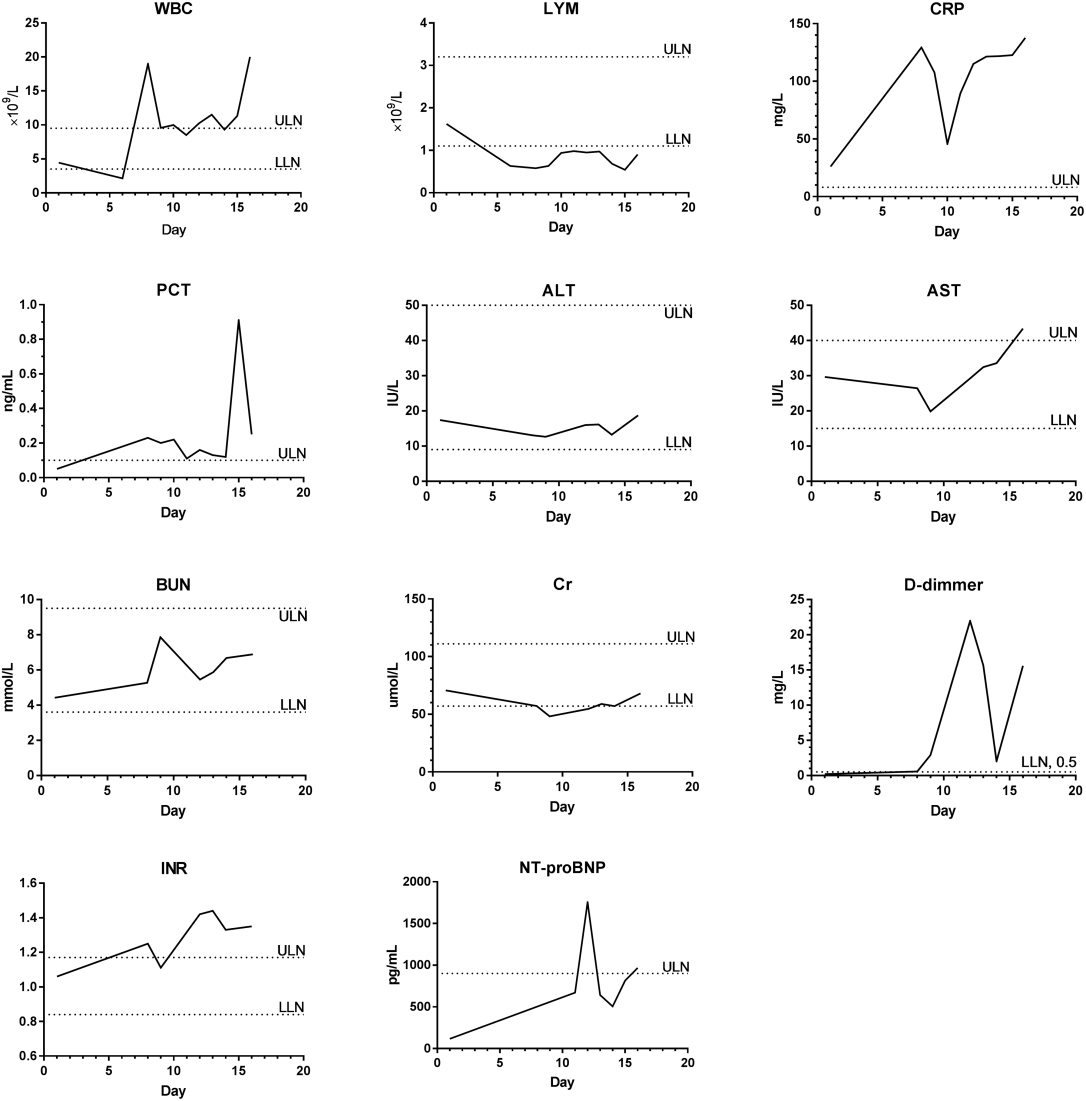
The dynamics of the main laboratory examinations of P6. WBC, White blood cell count; LYM, Lymphocyte count; CRP, C-reactive protein; PCT, Procalcitonin; ALT, Alanine aminotransferase; AST, Aspartate aminotransferase; BUN, Blood urea nitrogen; Cr, Creatinine; INR, International normalized ratio; NT-proBNP, N-terminal pro-B type natriuretic peptide; ULN, Upper limit of normal; LLN, Lower limit of normal.

**Figure 3:**
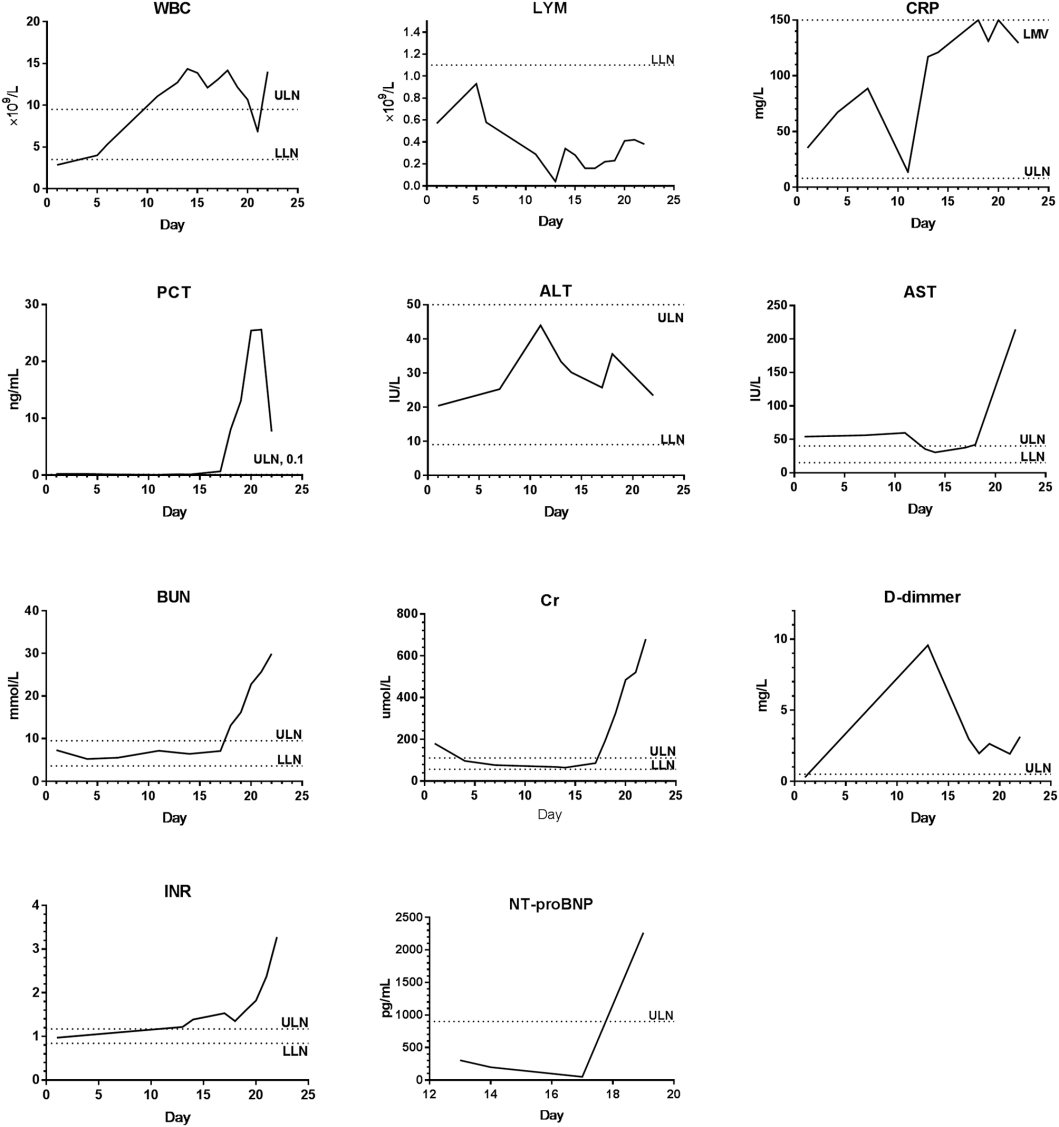
The dynamics of the main laboratory examinations of P7. WBC, White blood cell count; LYM, Lymphocyte count; CRP, C-reactive protein; PCT, Procalcitonin; ALT, Alanine aminotransferase; AST, Aspartate aminotransferase; BUN, Blood urea nitrogen; Cr, Creatinine; INR, International normalized ratio; NT-proBNP, N-terminal pro-B type natriuretic peptide; ULN, Upper limit of normal; LLN, Lower limit of normal.

The laboratory examination showed that 4 patients presented with decreased white blood cell counts, 3 with lymphocyte counts, and 5 with hyponatremia and hypokalemia. Four patients presented with liver dysfunction, and the main changes were an increase in alanine aminotransferase or aspartate aminotransferase. Renal dysfunction was noted in 3 patients and showed an increase of creatinine. Abnormal serum enzymatic results were found in 5 patients, mainly manifested by an increase in creatine kinase, lactate dehydrogenase (LDH), and α-hydroxybutyrate dehydrogenase (α-HBDH). Five patients had abnormal coagulation function, including the prolongation of prothrombin time, activated partial thromboplastin time and thrombin time, and the increase of fibrinogen. D-dimer, international normalized ratio, plasma protamine paracoagulation test were normal. Procalcitonin was increased in 1 patient, C-reactive protein increased in 5 patients, and erythrocyte sedimentation rate increased in 3 patients. One patient (P7) had cytomegalovirus and bacterial infection during the course of the illness, with significant increase in white blood cell count and body temperature exceeded 39°C (1 day), but the lymphocyte count continued to decrease. During the treatment of critically ill patients, continuous or progressive decrease in white blood cell and lymphocyte, deranged liver and kidney function, elevated coagulation function index, elevated C-reactive protein, and increased erythrocyte sedimentation rate were noted (Table 3). The dynamics of the main laboratory examinations of 2 critically ill patients (P6 and P7) were shown in Figures 2, 3.

**Table 3:** Radiographic and laboratory results of COVID-19 patients with gastrointestinal symptoms on admission.

**Variable**	**Normal range**	**P1**	**P2**	**P3**	**P4**	**P5**	**P6**	**P7**
Chest CT scan	Negative	Patchy ground glass opacities	Patchy ground glass opacities	Patchy ground glass opacities	Patchy ground glass opacities and partial consolidation	Patchy ground glass opacities with thickened interlobular septa	Patchy ground glass opacities	Patchy high-density shape
White blood cell count, ×10^9^/L	3.5–9.5	2.43 (↓)	2.99 (↓)	3.35 (↓)	5.08	4.40	4.44	2.86 (↓)
Neutrophil ratio, %	40–75	68.8	58.2	53.7	63.3	48.7	44.8	70.3
Lymphocyte ratio, %	22–50	22.6	32.1	37.0	28.8	36.2	36.5	19.9 (↓)
Lymphocyte count, ×10^9^/L	1.1–3.2	0.55 (↓)	0.96 (↓)	1.24	1.46	1.59	1.62	0.57 (↓)
Monocyte ratio, %	3–10	8.6	9.4	9.0	7.4	7.6	18.0 (↑)	9.8
Monocyte count, ×10^9^/L	0.1–0.6	0.21	0.28	0.30	0.38	0.33	0.80 (↑)	0.28
Fecal occult blood test	Negative	NA	Negative	NA	NA	NA	NA	Negative
Fecal transferrin test	Negative	NA	Negative	NA	NA	NA	NA	Weak positive
Fecal leukocyte test	Negative	NA	Negative	NA	NA	NA	NA	Positive
Potassium, mmol/L	3.5–5.3	3.63	3.35 (↓)	4.48	3.83	3.75	4.37	3.87
Sodium, mmol/L	137–147	134.49 (↓)	139.92	140.73	135.92 (↓)	139.03	136.42 (↓)	135.36 (↓)
Alanine aminotransferase, IU/L	7–40	18.06	20.90	9.50	64.18 (↑)	54.93 (↑)	17.39	20.10
Aspartate aminotransferase, IU/L	13–35	32.27	39.20 (↑)	13.15	43.29 (↑)	28.48	29.65	54.10 (↑)
Blood urea nitrogen, mmol/L	2.6–7.5	2.21 (↓)	5.15	2.61	4.81	2.82	4.42	7.31
Creatinine, μmol /L	41–73	52.24	48.70	57.34	80.22 (↑)	77.82 (↑)	70.42	180.30 (↑)
Creatine kinase, U/L	40–200	210.68 (↑)	34.00 (↓)	38.06(↓)	457.70 (↑)	93.78	54.10	293.00 (↑)
Lactate dehydrogenase, U/L	120–250	278.2 (↑)	165.0	172.0	265.5 (↑)	183.4	201.0	254.0 (↑)
α-hydroxybutyrate dehydrogenase, U/L	72–182	216.72 (↑)	133.20	126.35	179.27	124.48	127.84	182.50 (↑)
C-reactive protein, mg/L	0–8	11.83 (↑)	5.58	2.85	13.24 (↑)	20.71 (↑)	26.12 (↑)	35.26 (↑)
Procalcitonin, ng/mL	<0.1	<0.05	<0.05	<0.05	0.05	0.06	0.05	0.22 (↑)
Erythrocyte sedimentation rate, mm/h	0–20	21 (↑)	12	32 (↑)	12	18	19	75 (↑)
D-dimer, mg/L	0–0.5	0.03	0.34	0.08	0.03	0.03	0.19	0.28
International normalized ratio	0.84–1.17	1.03	0.84	0.97	0.87	1.06	1.06	0.97
Prothrombin time, sec	9–14	11.6	9.8	11.0	10.1	11.9	11.5	11.3
Activated partial thromboplastin time, sec	21–40	34.7	27.6	24.6	27.6	28.0	42.7 (↑)	97.1 (↑)
thrombin time, sec	8–14	11.4	16.9 (↑)	10.4	10.3	11.4	12.9	24.3 (↑)
Fibrinogen, g/L	2–4	3.40	2.87	6.17 (↑)	4.09(↑)	3.07	4.10 (↑)	3.49
plasma protamine paracoagulation test	Negative	Negative	Negative	Negative	Negative	Negative	Negative	Negative

*NA, not available; ↑, above normal range; ↓, below normal range*.

All patients received conventional antiviral, antibiotics, and Chinese patent medicine. Antiviral drugs included recombinant human interferon-α1b and lopinavir/ritonavir tablets. Chinese patent medicine included Lianhua Qingwen Capsule and Xuanfei Zhike mixture. Antibiotics included levofloxacin, moxifloxacin, and piperacillin/sulbactam, or combination of them if necessary. Montmorillonite powder was used in patients with diarrhea. Pantoprazole and metoclopramide were used in patients with severe nausea and vomiting. Those with obvious electrolyte disturbance and anorexia were given potassium, sodium and nutritional support. Three patients (P1, P6, and P7) developed progressive dyspnea during the disease progression and received respiratory support and low-dose short-term methylprednisolone. As of March 13, 2020, there were 5 patients who were recovered, and 2 patients (P6 and P7) have died. One month after discharge, the chest CT of P1 showed that scattered fiber lines were distributed in both lungs, but there were no clinical symptoms of the respiratory tract and digestive tract, and hematologic and nucleic acid tests were also negative. Positive signs were not observed in the remaining 4 patients, including clinical symptoms, chest CT scan, hematologic test, and nucleic acid test.

## Discussion

SARS-CoV-2 has the characteristics of strong contagion and rapid transmission (14). As of 24:00 on February 24, 2020, 77,658 confirmed cases of COVID-19 were reported in China, and nearly one thousand confirmed patients were found in neighboring countries such as Korea and Japan. Although China, especially Hubei Province, has adopted strict preventive and control measures, confirmed cases continue to appear. A comprehensive and in-depth understanding of SARS-CoV-2 has great practical significance, which can guide early detection, isolation, treatment of COVID-19, and control the source of infection and block the transmission, as well as reduce morbidity, severity, and mortality.

We established a cohort of 542 suspected and confirmed COVID-19 patients admitted to Xiangyang No. 1 People's Hospital affiliated to Hubei University of Medical by April 9, 2020. Among them, 142 patients have been confirmed. Although COVID-19 patients were characterized by fever and fatigue and respiratory symptoms such as cough, choking sensation in chest, shortness of breath, and dyspnea, they may present with other symptoms, such as gastrointestinal symptoms. Our study included 7 patients with gastrointestinal symptoms, accounting for 4.9% of all confirmed patients. Although the number of cases included in this study was small, these patients might reflect the clinical manifestations and disease progression of this special type of COVID-19 patients. All the 7 patients had fever, but a few of them had no respiratory symptoms or showed respiratory symptoms in the middle and late stages of the disease. The main clinical manifestations of these 7 patients were gastrointestinal symptoms. In China, all the hospitals have fever outpatient department after the SARS-CoV-2 outbreak. Thus, patients with atypical symptoms of COVID-19 can be examined further and thoroughly after clinical inquiry of their travel history to infectious regions. However, these patients may have a missed or delayed diagnosis if fever outpatient department is not available. Such a missed or delayed diagnosis may cause severe transmission of SARS-CoV-2 to others.

The first symptom of one patient (P1) was diarrhea, which was mainly manifested as watery stools (16 days), accompanied by intermittent hypothermia, whereas respiratory symptoms appeared at a later stage. One patient (P3) had anorexia (7 days) together with nausea (6 days), diarrhea, upper abdominal discomfort, and intermittent low fever, and no respiratory symptoms. Another patient (P2) had chief complaint of diarrhea and intermittent low fever, but the course of disease was characterized by anorexia, diarrhea, nausea and upper abdominal discomfort, and short-term cough, choking sensation in chest, and myalgia occurred during disease progression. Although fever or cough was the chief complaint in the other 4 patients, the main symptoms in the course of disease were gastrointestinal symptoms such as anorexia and diarrhea, and most of the diarrhea was watery stools. At admission, the physical examination of 7 patients showed no abdominal tenderness, rebound tenderness, Murphy's sign and abdominal rigidity. Based on the discussion above, we summarized that the clinical features of COVID-19 patients with gastrointestinal symptoms may be watery stools, anorexia, upper abdominal discomfort, nausea with low fever, and no obvious positive signs of the digestive tract.

Both SARS-CoV-2 and severe acute respiratory syndrome coronavirus (SARS-CoV) are β-type coronaviruses that are mainly transmitted through respiratory droplets and close contact (15). Angiotensin-converting enzyme 2 (ACE-2) is the receptor critical for mediating SARS-CoV entry into host cells (2, 16). ACE-2 receptor mainly exists in alveolar type II (ATII) cells, which may be the reason for the lung injury and respiratory symptoms caused by COVID-19. However, recent studies had shown that ACE-2 receptors were also highly expressed in esophageal stratified epithelial cells, and in ileum and colon resorbable epithelial cells (17). Such a high expression of ACE-2 in the digestive system supports our clinical observation that patients with COVID-19 might have initial clinical symptoms from the digestive tract (18). It is still unclear how SARS-CoV-2 enters the gastrointestinal tract. Some researchers have indicated that immune cells produced by infected lung cells can cause gastrointestinal infections and trigger gastrointestinal-related symptoms (19). Some previous studies isolated a larger number of SARS-CoV-2 from stools of patients with COVID-19 and they believed that SARS-CoV-2 may have fecal-oral transmission (14, 20, 21). Based on the finding of a large amount of SARS-CoV-2 virus in the stool, it was suggested that SARS-CoV-2 was less likely to be infected by cell debris derived from the respiratory tract, and more likely due to replication in the digestive tract (22). Recent data from environmental samples suggested that viral shedding in stool could be a potential route of transmission after positive findings from toilet bowl and sink samples (23). However, there is no direct evidence of fecal-oral transmission, which call for further studies to explore this knowledge gap. It is recommended by WHO that hand hygiene, separate eating, keeping toilets clean, and proper fecal management should become key measures for prevention and control of COVID-19 (21).

The first CT examination of 7 patients showed typical COVID-19 imaging findings, such as multifocal nodules in the bilateral, subpleural lung parenchyma, patchy ground-glass changes in the subpleural, crazy-paving pattern, white lung appearance and air bronchograms (24–26). One week later, it was found that the lesions of the lung lobes increased and enlarged, involving bilateral lungs or multiple lung lobes, and some solid changes and fibrous cord lesions being visible. CT examination 2 weeks later showed that the ground-glass lesions and nodules were absorbed and decreased compared with the previous one, and the fibrous focus of most patients was significantly increased (27). For critically ill patient (P6) X-ray showed that fibrosis of the lungs became worse with white lung appearance. We dynamically evaluated chest CT images and found that all patients had large-scale and severe lung injury, indicating that patients with COVID-19 gastrointestinal symptoms also had obvious lung injury, and may be more severe than ordinary COVID-19 (9).

The results of laboratory examination showed a similar observation to previous reports (10). During the course of disease, all patients had increased C-reactive protein, accelerated erythrocyte sedimentation rate, hypoproteinemia, and mild electrolyte disturbances. Most patients had a lower count of white blood cells and lymphocytes. Persistent lymphopenia was found in severe COVID-19 patients (4, 9). In the middle and late stages, there was still a decrease in lymphocytes, which might be related to the poor consumption and response ability of the immune system (10). C-reactive protein continued to increase and was >60 mg/L in severe patients, indicating that the body had a severe inflammatory response. The sustained increase in LDH and α-HBDH in severe patients indicated a persistent cell damage, which may be caused by damage to the digestive organs such as the liver. Therefore, the prognosis of these patients was poor. Persistent lymphopenia, increased C-reactive protein, and persistently elevated LDH and α-HBDH may be signals of COVID-19 progression.

Three of the seven patients in this study were severe cases, and 2 of them were critically ill. Severe patients initially had choking sensation in chest, dyspnea, and progressive decrease in oxygen saturation. As the disease progressed, multiple organ dysfunctions occurred, eventually leading to multiple organ failure or even death. The final clinical outcome was recovered in 5 patients and dead in 2 patient. Analysis of disease severity and prognosis together with CT imaging performance showed that the severity of illness was consistent with CT imaging. We can evaluate the severity and prognosis of the disease through CT imaging by dynamic examination (13). For patients with COVID-19, CT scans can be used to detect lung lesions. However, there is a lack of available measures to detect digestive system lesions. Moreover, it is unclear how the damage to the digestive system affects the prognosis of the disease. Therefore, more research is needed to further elucidate the digestive system changes in patients with COVID-19.

Given that there is currently no effective treatment for COVID-19, antiviral, antibiotic, Chinese patent medicine, and supportive treatment is still the main option in clinical treatment (10, 28, 29). The antiviral treatment in this study included: recombinant human interferon-α1b aerosol inhaled, with or without oral lopinavir/ritonavir tablets. Levofloxacin, moxifloxacin or piperacillin/sulbactam were used for antibiotic treatment and can be combined in severe cases. Chinese patent medicine has also been widely used in clinical practice, such as Lianhua Qingwen Capsule and Xuanfei Zhike mixture (8). In most patients, symptoms will be relieved within 7 days of treatment, and chest CT lesions can be absorbed around 14 days. For patients with diarrhea, the symptoms disappeared after 3–5 days of treatment with montmorillonite powder and nutritional support treatment. Except for 2 elderly patients with poor prognosis, the rest recovered quickly and the prognosis was good.

## Conclusions

This study described seven COVID-19 patients with gastrointestinal symptoms and provided a reference for disease management and prevention. COVID-19 patients with gastrointestinal symptoms are relatively rare and may be misdiagnosed. These patients have severe disease and are associated with a poor prognosis. The underlying mechanisms for the development of gastrointestinal symptoms need to be clarified in future studies.

## Data Availability Statement

The original contributions presented in the study are included in the article/Supplementary Material, further inquiries can be directed to the corresponding authors.

## Author Contributions

BP, JJ, and X-TZ designed the study and reviewed the manuscript. J-WA, YW, and L-YL collected data. QH and HZ performed statistic analysis. J-WA, HZ, YW, and NW summarize the data and drafted the manuscript. All authors were responsible for final approval of the version to be published.

## Conflict of Interest

The authors declare that the research was conducted in the absence of any commercial or financial relationships that could be construed as a potential conflict of interest.
