# Supplementary material for: Clinical Characteristics of COVID-19 Patients With Gastrointestinal Symptoms: An Analysis of Seven Patients in China
Source: Front Med (Lausanne). 2020 Jun 9;7:308. doi: 10.3389/fmed.2020.00308 (PMC7325892; doi:10.3389/fmed.2020.00308)
Supplement: Supplementary file 1 [file Data_Sheet_1.pdf]

Supplementary material

Figure S1. The typical progression of radiology in COVID-19 patients.

Figure S2. The radiological images of P2, P3, P4, P5, and P6.

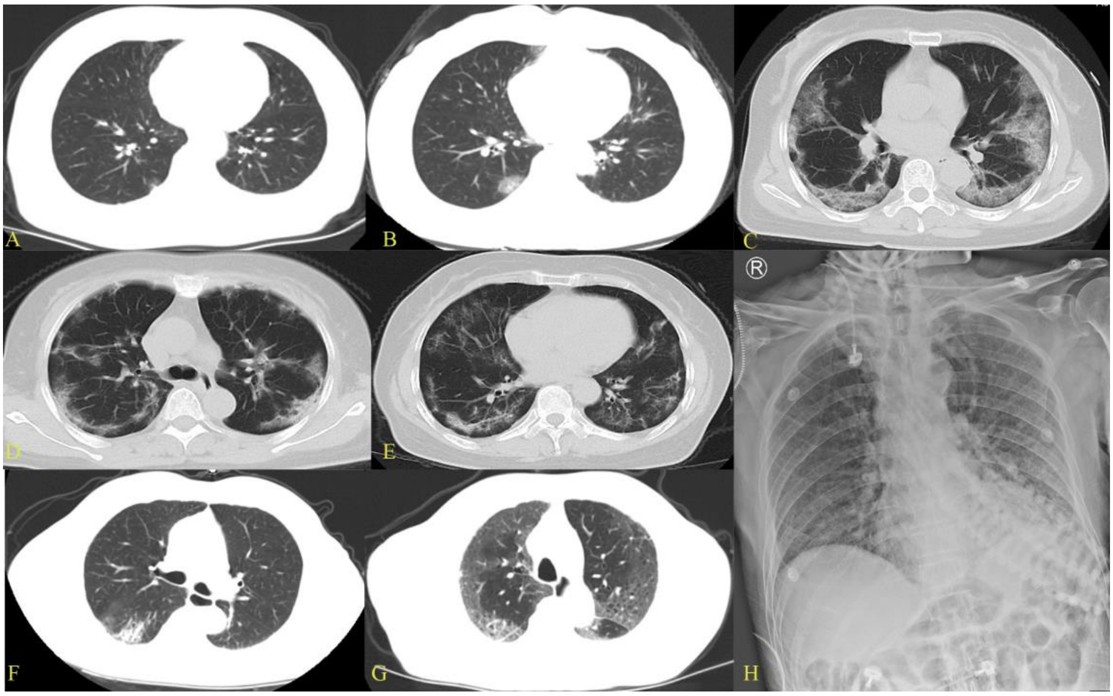

Figure S1. The typical progression of radiology in COVID-19 patients.

P1: 54-year-old female, with onset of January 24, 2020 and admission on January 31, 2020 (A-E).

P7: 75-year-old male, with onset of January 20, 2020 and admission on January 24, 2020 (F-H).

(A) January 26, patchy ground glass opacities under the pleura in the right lower lobe. (B) January 29, patchy ground glass opacities and partial consolidation under the pleura in the right lower lobe. (C) February 5, multiple patchy ground glass opacities and partial fibrosis in the peripheral pleura of bilateral lungs. (D) February 12, most of the ground glass opacities were absorbed, with multiple fiber strands remaining. (E) February 20, ground glass opacities were further absorbed, and a few fiber strands were visible. (F) February 23, patchy high-density shape of the right lower lobe. (G) February 29, newly developed patchy ground glass opacities in bilateral lungs, with thickening of interlobular septa. (H) February 7, the chest X-ray showed patchy high-density shape in bilateral lungs.

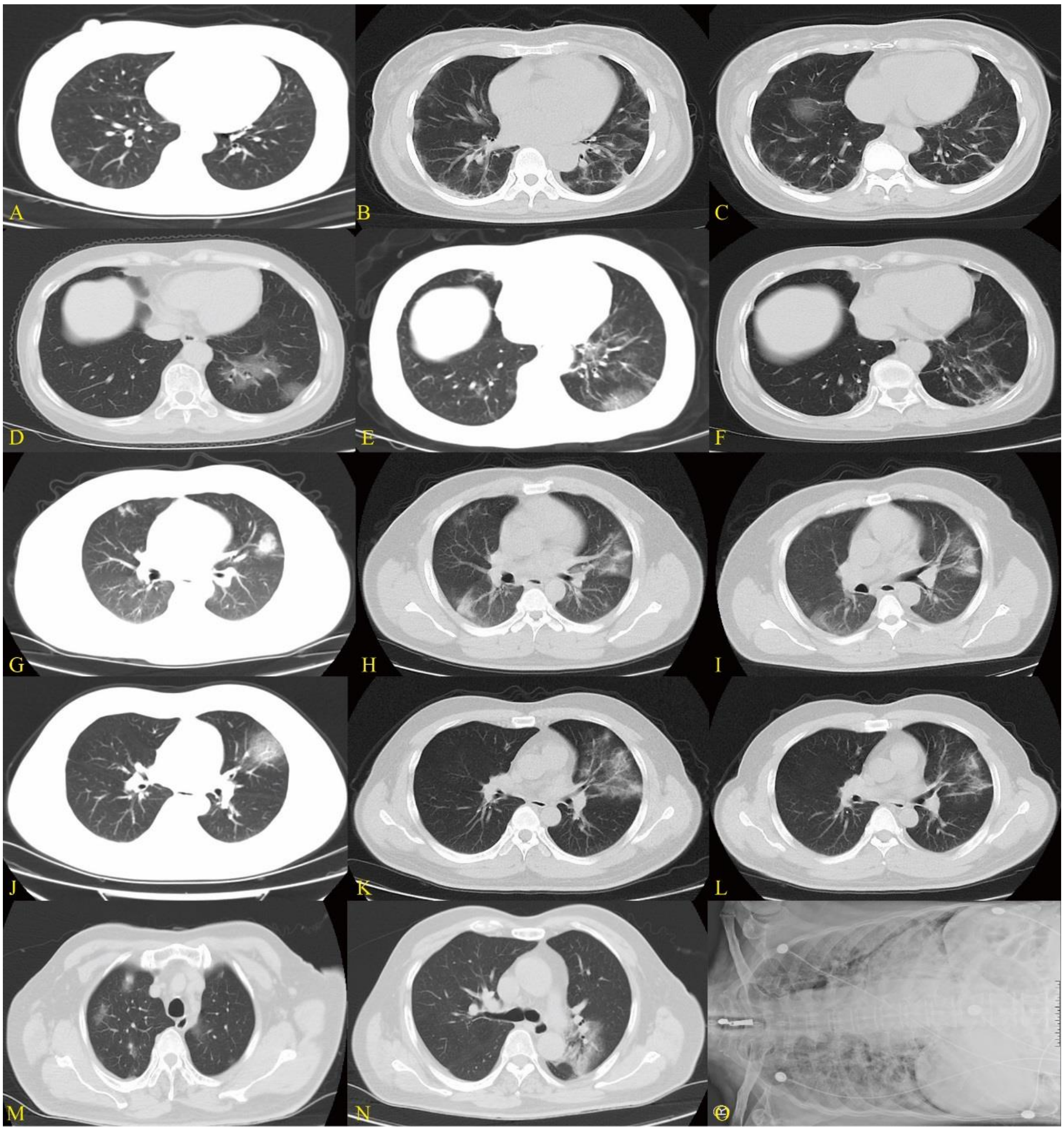

Figure S2. The radiological images of P2, P3, P4, P5, and P6.

P2: 48-year-old female, the chest CT showed multiple patchy ground glass opacities (A), multiple patchy ground glass opacities and fiber strands (B), and ground glass opacities were absorbed and few fiber strands remained (C).

P3: 50-year-old female, the lesions were concentrated in the left lower lobe, and patchy ground glass opacities were distributed along the subpleural and bronchovascular bundle (D). The ground glass opacities were absorbed, with fiber strands remaining (E and F).

P4: 35-year-old male, patchy ground glass opacities and partial consolidation were distributed in the right middle lobe and lingular segment of the left upper lobe (G). Patchy ground glass opacities and partial consolidation in bilateral lungs, suggesting disease progression (H). The patchy ground glass

opacities were absorbed, and a few fiber strands were visible (I).

P5: 42-year-old male, the lesions were concentrated in the left upper lobe, and patchy ground glass opacities with thickened interlobular septa were seen under the pleura (J). Patchy ground opacities and partial consolidation in the left upper lobe, suggesting disease progression (K). The patchy ground glass opacities were absorbed, and a few fiber strands were visible (L).

P6: 75-year-old male, the lesions were concentrated in the upper lobe of bilateral lungs, and patchy ground glass opacities were distributed along the subpleural and bronchovascular bundle (M). Patchy ground glass opacities and partial consolidation in the left upper lobe, suggesting disease progression (N). Multiple patchy high-density shape in bilateral lungs (O).
